# Isotope Effects on Chemical Shifts in the Study of Hydrogen Bonds in Small Molecules

**DOI:** 10.3390/molecules27082405

**Published:** 2022-04-08

**Authors:** Poul Erik Hansen

**Affiliations:** Department of Science and Environment, Roskilde University, Universitetsvej 1, DK-4000 Roskilde, Denmark; poulerik@ruc.dk

**Keywords:** hydrogen bonding, isotope effects on ^13^C chemical shifts, tautomerism, hydrogen bond energy, theoretical calculations

## Abstract

This review is giving a short introduction to the techniques used to investigate isotope effects on NMR chemical shifts. The review is discussing how isotope effects on chemical shifts can be used to elucidate the importance of either intra- or intermolecular hydrogen bonding in ionic liquids, of ammonium ions in a confined space, how isotope effects can help define dimers, trimers, etc., how isotope effects can lead to structural parameters such as distances and give information about ion pairing. Tautomerism is by advantage investigated by isotope effects on chemical shifts both in symmetric and asymmetric systems. The relationship between hydrogen bond energies and two-bond deuterium isotope effects on chemical shifts is described. Finally, theoretical calculations to obtain isotope effects on chemical shifts are looked into.

## 1. Introduction

Hydrogen bonding, both inter- and intramolecular, has profound effects on the properties of small molecules, reactivity, pK_a_ values, polarity, solubility and with that, e.g., penetration of membranes and biological effects in general. Most drugs and bioactive molecules are small molecules and often depend on binding to receptors partly via hydrogen bonds [1,2]. Related to intramolecular hydrogen bonding is tautomerism. One of the effective tools in the study of hydrogen bonding is isotope effects on NMR chemical shifts. Both primary and secondary isotope effects may be used. The most common isotope is ^2^H, deuterium (D), but also ^13^C, ^15^N and ^18^O as well as more rare isotopes are used. For observation of secondary isotope effects, the most common nuclei are ^1^H, ^13^C, ^15^N, ^19^F and ^18^O. In case of primary isotope effects, the pair ^1^H, ^2^H is mostly used. The intrinsic isotope effects are defined as:

Primary: ^P^ΔH(D) = δ(H) − δ(D); 

Secondary: ^n^ΔX(h) = δX(l) − δX(h).

l = light, h = heavy and X is the nucleus under investigation, n is the number of covalent bonds between the isotope and the investigated nucleus. For intramolecular hydrogen bonded cases, the isotope effects may be transmitted via the hydrogen bond. In the cases in which to opposite sign convention has been used, the sign is changed and the number is marked with an asterisk.

Deuterium isotope effects can be related to hydrogen bond strength and to hydrogen bond energies. Isotope effects are very useful in the equilibria of symmetric systems (lifting of degeneracy). They respond to nearby charges.

Most of the studies were performed in the liquid state, but isotope effects on chemical shifts can also in favorable cases be studied in the solid state.

The present review will primarily cover the last ten years. Other reviews that cover the subject are [3,4,5,6].

## 2. Techniques

### 2.1. Liquid State

As hydrogen bonding is the subject, the most relevant protons to exchange are those of OH, NH or SH groups. Deuteration can easily be achieved either by shaking with D_2_O in, e.g., chloroform or methylene chloride and subsequent drying with water-free sodium sulphate or by dissolution in CH_3_OD followed by evaporation. In case of exchange of CH protons this depends very much on the compound. An example is imidazolium acetate liquid ionic liquids which have been used to catalyze H-D exchange in 2-alkanones using CD_3_OD as deuterium source. Long-range isotope effect on ^13^C chemical shifts is measured [7]. For a different case see Section 3.1. Introduction of ^18^O may be obtained by exchange, whereas ^15^N usually requires a synthetic scheme. In the case of deuteration, a degree of deuteration of ~70% is desirable as some lines in the spectrum may become broad due to couplings to deuterium and a clear-cut difference in intensities between molecules with H and those with D is required in order to determine the signs of the isotope effects. As slow exchange is a prerequisite to measure isotope effects directly, low temperature may be necessary. For examples of very low temperature, see references in this paper and previous papers by the Limbach-Tolstoy group [6]. Cooling down may not only slow down the XD exchange but may also lead to observation of isotope effects of single rotamers as seen in, e.g., 2,6-dihydroxyacylaromatics (see Section 3.5). Exchange can be slowed down by use of a hydrogen bonding solvent such as DMSO [8]. In the case of ammonium ions, exchange may be slowed down by acidification.

For compounds only soluble in water, the deuterium isotope effects can be determined by recording spectra with varying ratios of H_2_O/D_2_O followed by extrapolations to 0 and 100% D_2_O.

Isotope effects can in most cases be determined by simple 1D NMR spectra. However, for ^1^ΔN(D) isotope effects it is an advantage to use indirect techniques. D.F. Hansen et al. have developed a ^13^C-detected ^15^N double-quantum NMR experiment [9]. In cases with exchange, the HISQC technique is useful [10].

**Figure 1:**
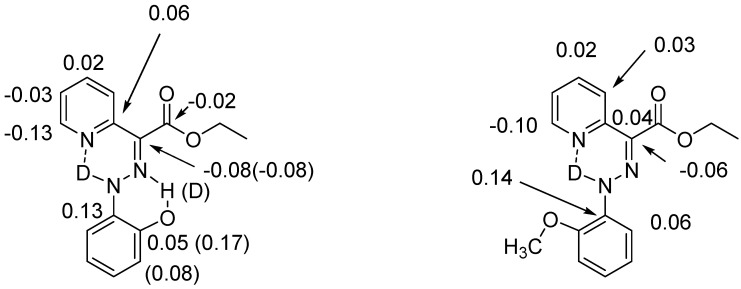
Deuterium isotope effects on ^13^C chemical shifts. Values in brackets are due to the deuterium in brackets. Data from Ref. [11].

Assignment of isotope effects in molecules with more than one site of e.g., deuteration can be achieved by utilizing different degrees of incorporation or different rates of incorporation. Examples are in β-diketones and in enaminones. In these cases, the hydrogen at the central carbon exchange much more slowly than the XH protons. In other cases, either a spatial separation or a comparison with similar compounds with fewer sites of deuteration can be used (see Figure 1)

### 2.2. Solid State

Isotope effects on chemical shifts can in principle also be measured in solid-state magic angle spinning spectra. However, as the lines typically are broad only large isotope effects can be resolved. The isotope effects analyzed recently are deuterium isotope effects on ^13^C chemical shifts in a tautomeric case as isotope effects may be large in tautomeric systems. An example is the pyridoyl benzoyl β-diketones see Section 4. Additionally, deuterium isotope effects at ^15^N chemical shifts could be observed in the triclinic phase of the complex between pentachlorophenol and 4-methylpyridine (Section 4). Another recent case is deuterium isotope effects on ^15^N chemical shifts of ammonium ions (see Section 3.2). For early examples, see Refs. [5,6].

## 3. Static Cases

### 3.1. Ionic Liquids

Over the past two decades, ionic liquids have become very versatile and “green” solvents in which hydrogen bonding is also important. This has spurred research into the properties, but also led to new types of ionic liquids.

**Figure 2:**
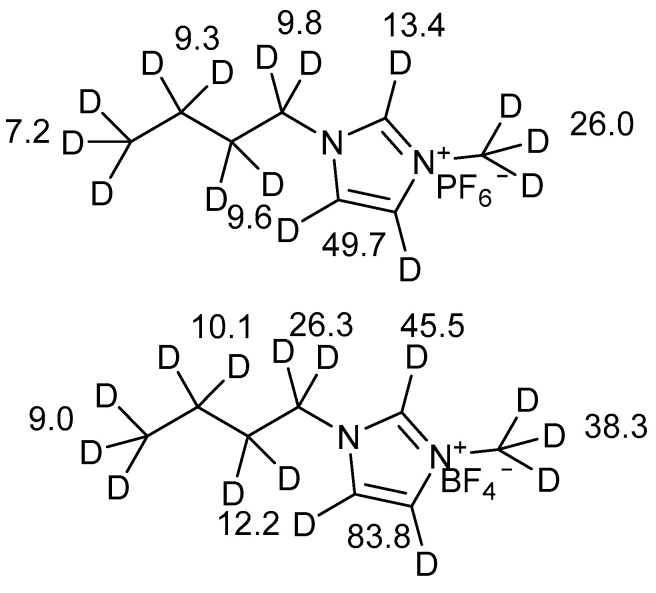
1-n-butyl-3-methylimidazolium PF_6_^−^ on top and the BF_4_^−^ below. The numbers are the deuterium isotope effects from that particular set of deuterium seen at the fluorine signals of the counter ions. In the case of BF_4_^−^, the effects caused by D-2 and D-3 are actually also caused by D-5. Data for the aliphatic deuterations from Ref. [12] and those caused by deuteration at C-2, C-3 and C-5 from Ref. [13].

Moyna et al. [12] studied the deuterium isotope effects at ^19^F chemical shifts of the counter ions PF_6_^−^ and BF_4_^−^ caused by deuterium of deuterated side-chains of 1-n-butyl-3-methylimidazolium (Figure 2).

The effects caused by deuteration at the aliphatic chain are largest closest to the ring. The effects correlate with the change in polarization of the C-H bonds. The effects are largest for BF_4_^−^ as the electron density is largest at the fluorines of this counter ion.

**Figure 3:**
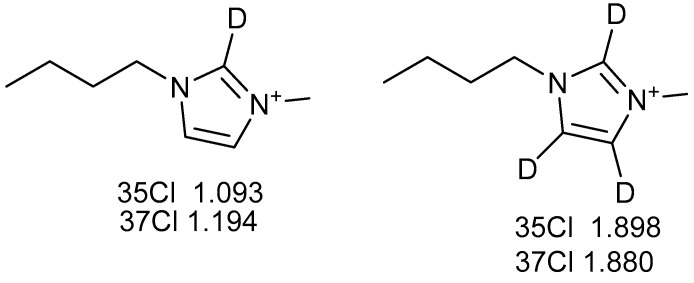
Numbers are deuterium isotope effects observed at the Cl resonance caused by the deuterium. Uncertainties ~0.1 ppm. Data from Ref. [14].

In a similar vein, deuterium isotope effects have been observed at the Cl^−^ chemical shifts when Cl^−^ is the counter ion [14] (Figure 3).

**Figure 4:**
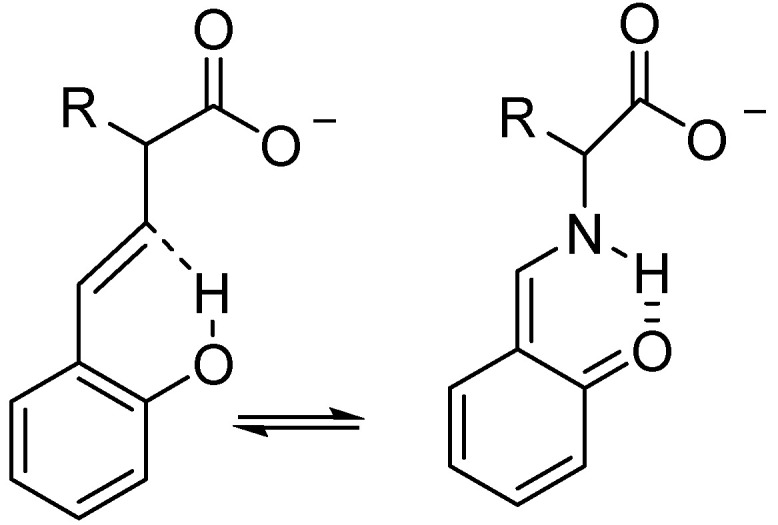
Ionic liquids based on amino acids.

Combined *o*-hydroxy Schiff bases and ionic liquids based on amino acids (Figure 4) showed based on ^2^ΔC-2(D) isotope effects that the COO^−^ group stabilizes the NH-form [15].

**Figure 5:**
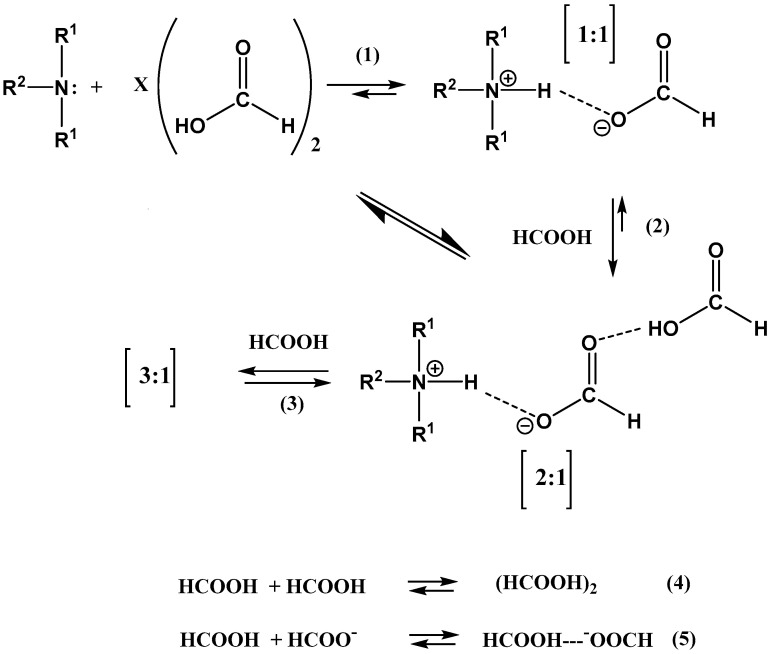
Suggested reaction scheme for the formation of the “ionic liquid”. Reprinted with permission from Ref. [17]. Copyright 2016 American Chemical Society.

In the reinvestigation of the diisopropylethylammonium formate, it was found that the 1:1 complex claimed by Anouti et al. [16] could not be reproduced. A more complex scheme was suggested (Figure 5) [17].

Primary deuterium isotope effects at the OH and NH^+^ resonances at 243 K were measured as 2.24 and 0.34 ppm, respectively. These values dropped to 1.08 and 0.30 ppm at 193 K. The mere fact that the OH resonance could be observed and with that, the primary isotope effect, proves that this is not a 1:1 complex. A study of the acetic acid dimer gave a primary deuterium isotope effect of 0.3 ppm and that of the acetic acid-acetate dimer 0.6 ppm [18]. It is obvious, based on an analogy with that result, that the OH(D) primary isotope effects are partly equilibrium isotope effects (Section 4). On the other hand, the NH^+^ is hydrogen bonded to the monomeric or to the dimeric acetate ion in the same fashion.

### 3.2. Ammonium Ions

**Figure 6:**
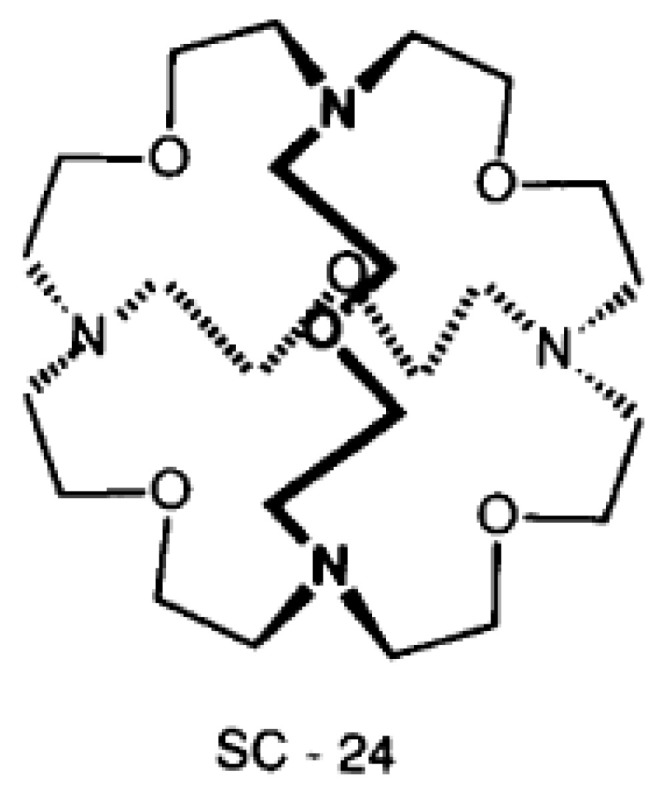
Structure of SC-24.

Ammonium ions and alkyl ammonium ions play an important role in biology [19,20]. Attempts to mimic enzymes were carried out by Lehn et al. [21] An example is SC-24 (Figure 6). Other confinements have been investigated recently [22].

Arginine showed deuterium isotope effects at the η nitrogen, 0.307 ppm measured in a H_2_O/D_2_O mixture 1:1. Due to the technique used it is an average for the two Nη nitrogen obtained, which means that the measured isotope effect is close to one half of ^1^ΔN(D). The values served as a reference for non-interacting residues in lysozyme [9].

Platzer et al. [23] measured deuterium isotope effects at the side-chain nitrogen of protonated and non-protonated lysines and argines being part of a tripeptide, acetyl-Gly-X-Glyamide. For the side-chain nitrogen of lysine, the values were 1.05 ppm for ND_3_^+^ and ~1.9 ppm for ND_2_. For arginines, the value for the ε nitrogen in the protonated state was 1.0 ppm and 1.4 ppm for the η nitrogen. In this case, it is not a pure one-bond isotope effect as long-range effects are present. In the case of lysines, the very different values between the protonated and non-protonated cases may be used to estimate the protonation state of lysines.

**Figure 7:**
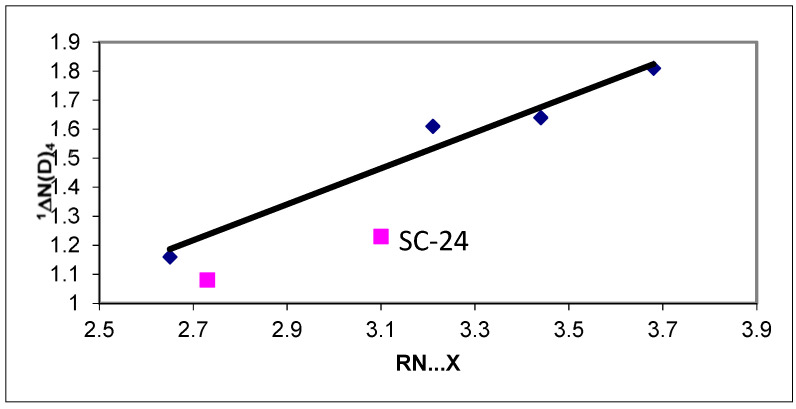
A plot of ^1^ΔN(D)_4_ vs. the heavy atom distances of halide ammonium ion salts. The second square is data for water. Data from Ref. [24].

In Figure 7 is shown a plot of ^1^ΔN(D)_4_ vs. the heavy atom distances of halide ammonium ion salts, I^−^, Br^−^, Cl^−^ and F is shown^−^. Marked with squares are data points for SC-24 and water. It is seen, that as the distance decreases, the one-bond deuterium isotope effect decreases. [24] A similar trend is found from theoretical calculations. [25] Furthermore, it is found that water is more effective than the halide ions. For SC-24, the one-bond deuterium isotope effects were found to be independent of the counter ions [26].

The two-bond deuterium isotope effects for complexes with 18-crown-6, 18-crown-6(COOH)_4_, dicylohexano 18-crown-6, kryptofix’s 2.2.2, 2.2.1 and 5, SC-24 and the ionophore and nonactin, are negative. The two-bond deuterium isotope effects are roughly proportional to the NH chemical shifts. The higher the NH chemical shifts, the more negative the two-bond deuterium isotope effects. In other words, the more negative the stronger the hydrogen bond. For SC-24, 18-crown-6(COOH)_4_ and nonactin, the two-bond deuterium isotope effects on ^1^H chemical shifts are independent of the counter ion. For the others, the more negative values are usually found for Cl^−^ rather than for NO_3_^−^ or I^−^ counter ions [27].

**Figure 8:**
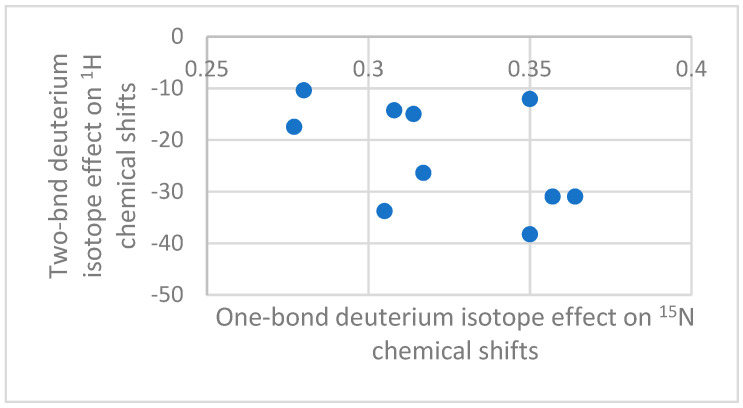
A plot of ^2^ΔH(D) vs. ^1^ΔN(D). Data from Ref. [26].

A plot of ^2^ΔH(D) vs. ^1^ΔN(D) shows very little correlation (Figure 8). It has been suggested that the ^15^N chemical shifts and with that the one-bond isotope effects depend on orbital overlap with the counter ion, whereas the two-bond deuterium isotope effects on ^1^H depend on electric field effects. In both cases, the isotope effect monitors ion pair formation.

### 3.3. Enaminones and Similar Compounds

**Figure 9:**
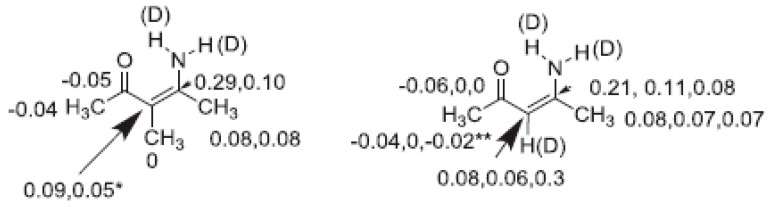
Deuterim isotope effects on 13C chemical shifts. * The central line is broad, so no isotope effect could be measured. ** Assignment tentative. Reprinted with permission from Ref. [29]. Copyright 2019 Elsevier.

**Figure 10:**
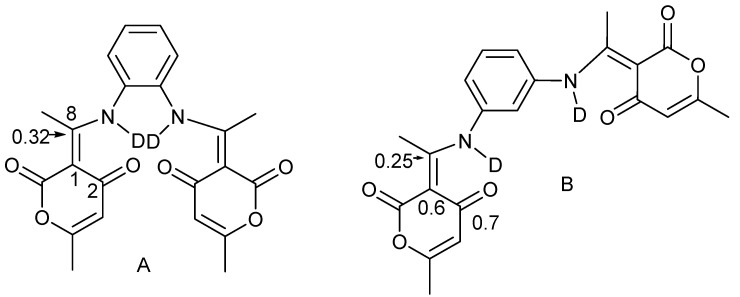
Two-bond deuterium isotope effects on ^13^C chemical shifts in ppm of phenylenediamine derivatives of dehydracetic acid deuterated. (**A**) Derivative based on *o-*phenylene diamine; (**B**) Derivative based on m-phenylene diamine Data from Ref. [28].

Data from the simple compounds such as those of Figure 9 may serve as reference points for more complex systems such as those of phenylene diamine derivatives of dehydracetic acid (Figure 10) [28]. The isotope effects in A are line with a non-tautomeric system, whereas those of B can only be explained by assuming a tautomeric equilibrium. 

**Figure 11:**
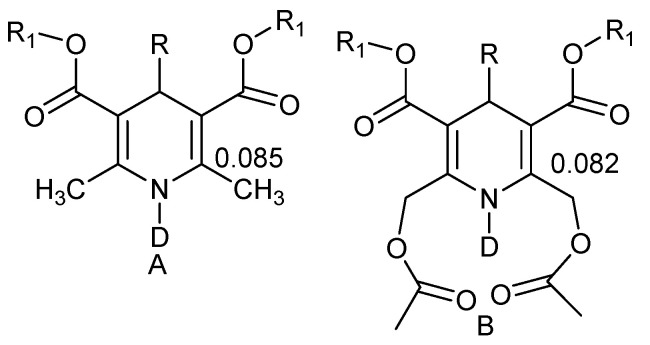
Two-bond deuterium isotope effects on ^13^C chemical shifts of 1,4-dihydropyridines. (**A**) Without intra-molecualr hydrogen bond; (**B**) with intramolecular hydrogen bond From Ref. [30].

Isotope effects have been measured in 1,4-dihydropyridines (Figure 11). The finding that the deuterium isotope effects on ^13^C chemical shifts are very similar in derivatives A and B [30] seems to show that the potential hydrogen bonding in B is weak.

### 3.4. Dimers and Trimers

**Figure 12:**
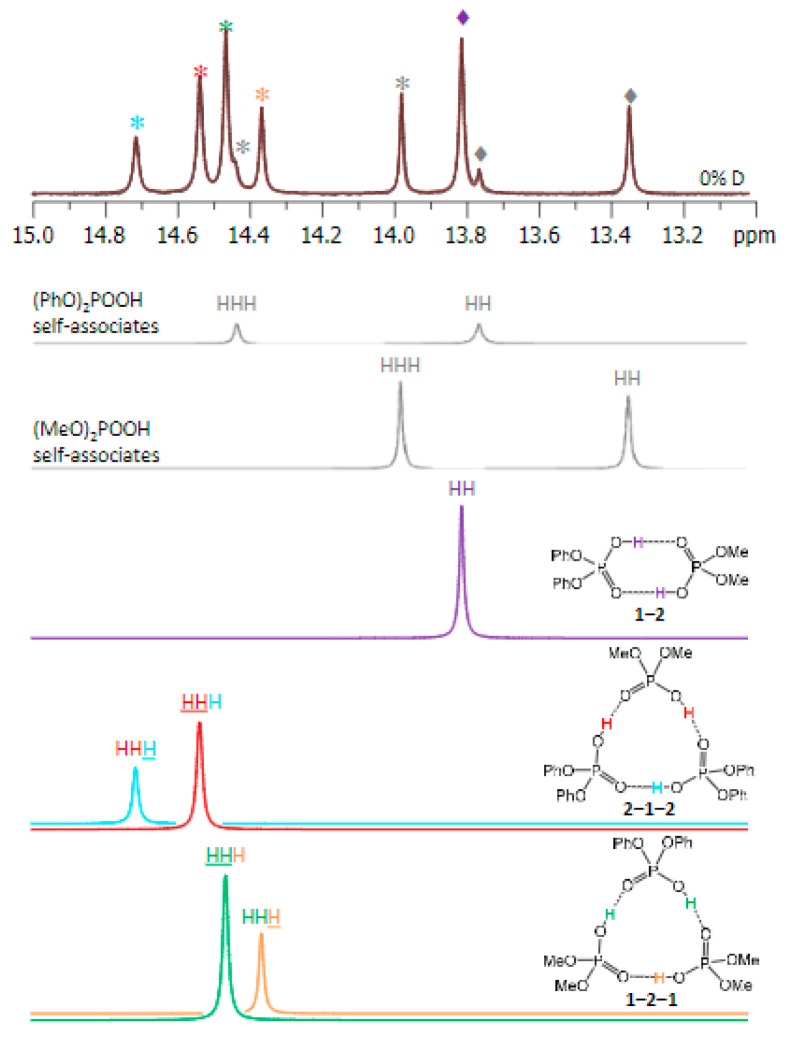
High-frequency part of the ^1^H NMR spectrum of partially deuterated (OH/OD, 57%D) of a mixture of diphenylphosphoric and dimethylphospinic acid in CDF_3_/CDF_2_Cl at 100 K. Trimers are marked with asterisks and dimers with diamonds. Reprinted from Ref. [32].

Tolstoy et al. in a very elegant way have used isotope effects on chemical shifts to determine the size of self-associated dimethylphosphinic, diphenylphosphoric acid, phenylphosphinic acid and bis(2,4,4-trimethylpentyl)phosphinic acid [31]. This has been extended to investigate heterodimers and heterotrimers of phosphinic and phosphoric acids (see Figure 12) [32]. Using the same technique, it could be proven that dimethylarsenic acid forms cyclic dimers in solution with two equivalent strong hydrogen bonds [33].

### 3.5. Miscellaneous

**Figure 13:**
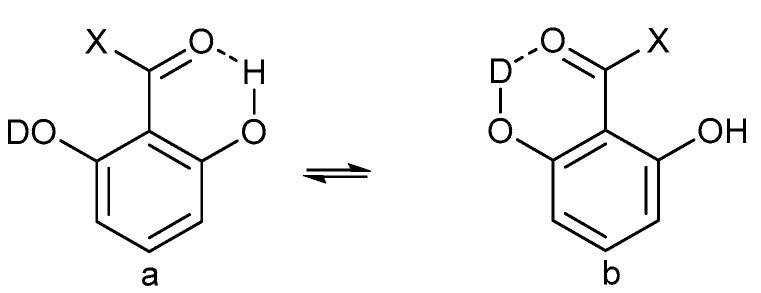
Equilibrium between monodeuterated 2,6-dihydroxyacylaromatics. X=H, CH_3_ or OCH_3_.

The equilibrium in the system shown in Figure 13 was originally determined using deuterium isotope perturbation techniques [34] and later calculated [35]. Xu et al. [36] investigated the similar system, 2,4-dihydroxybenzaldehyde measuring integrals and found that the deuterium prefers the non-hydrogen-bonded bond OH-4. O´Leary [37] analyzed the 2,6-dihydroxybanzaldehyde system in terms of vibrations and found that the high-frequency modes resulted in a Keq less than one, whereas the low- and medium-frequency modes resulted in a Keq > 1.

Deuterium isotope effects at C-2 at 1,1,1,3,3,3-hexafluoro-2-propanol-d_2_ have been investigated in CDCl_3_ and trimethylamine. The isotope effects were 0.364 and 0.341 ppm, respectively [38]. The authors ascribed the difference to complex formation, but the difference is very small and if anything in the wrong direction.

**Figure 14:**
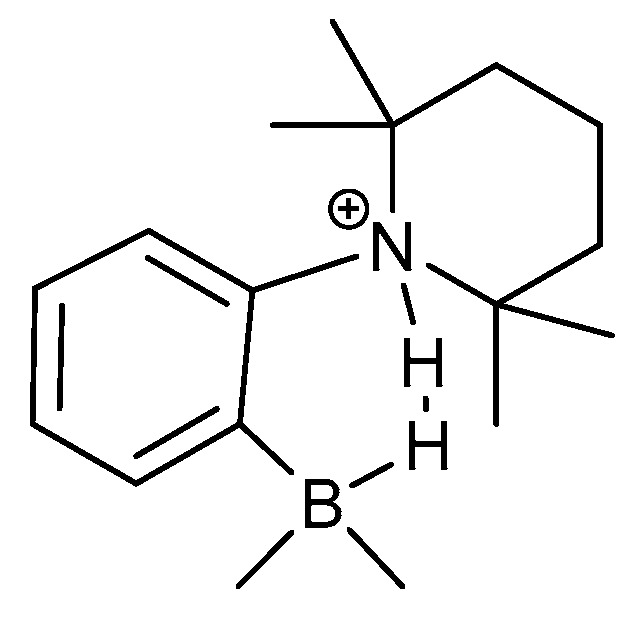
Structure of NHHB. The two hydrogens of the dihydrogen bond are selectively deuterated. From Ref. [39].

Schulz et al. [39] studied primary isotope effects in 1-N-TMPH-CH_2_-2[HB(C_6_F_5_)_2_]C_6_H_4_ (NHHB) (Figure 14). The molecule shows a strong dihydrogen bond. Deuteration of the NH proton led to a primary deuterium isotope effect of 0.56 ppm, whereas deuteration of the BH hydrogen did not lead to an isotope effect. The isotope effect of 0.56 ppm could indicate a double-well hydrogen bond potential [40]. However, the authors argued for a single-well potential, but they did not explain why the other effect was zero.

A xenon molecule in a deuterated hydrogen bond network of β-hydroquinones crystal shows an isotope effect of 2.4* ppm at 298 K and 2.6 ppm at 333 K. This effect is rather small considering the chemical shift range of Xe [41]. The effect is similar to that found in water/heavy water of 3.92* ppm. CH_3_OD gives an isotope effect of the opposite sign.

### 3.6. Cooperativity

Hydrogen bonded systems with two hydrogen bond donors to the same acceptor e.g., 1,8-dihydroxyanthraquinones or the monoanion of 1,8,9-trihydroxyanthracene [42] can give rise to cooperativity. A second situation seen in Figure 12 is the trimers of phosphoric, phospinic acids and a third situation seen is the dimers of carboxylic acids (for carboxylic acids encapsulated see Section 4). For the monoanion of 1,8,9-trihydroxyanthracene the primary isotope effects were −0.2 ppm in both DMSO-d_6_ and in 90%H_2_O/10% DMSO-d_6_. The degree of deuteration was 50% [42]. The finding of the same effect in those two solvents again showed that the hydrogen bond was strong enough not to be perturbed by the solvent. In the cyclic trimers both cooperative and anti-cooperative effects may be found. In the cooperative case, the X-D bond is weakened and the corresponding XdH bond is strengthened. The opposite is true for the anti-cooperative case [31]. In the cyclic trimers of phosphoric acids, cooperative effects are found [31]. In the case of hetero trimers of phosphinc and phosphoric acids, anti-cooperative effects are found probably due to steric factors [32]. For the trimers of dimethylarsinic acid, cooperativity is found [33].

## 4. Tautomerism

**Figure 15:**
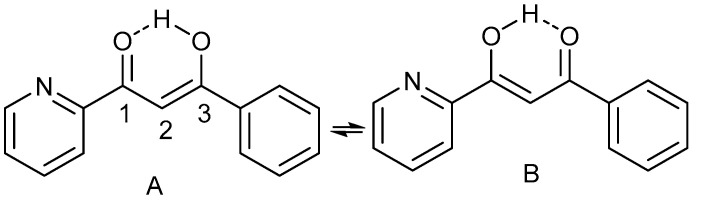
Tautomeric equilibrium illustrated by a β-diketone. The mole fraction of the B tautomer is x.

The use of isotope effects to investigate tautomerism has been treated in several reviews. [3,4,5] A key point is the observation in non-symmetrical systems of isotope effects on chemical shifts that they consist of both an intrinsic and an equilibrium contribution. A classic case is that of β-diketones, illustrated in Figure 15.

The equilibrium isotope effects can be formulated as seen in Equations (1)–(3).

^n^ΔX(D)_int_ = (1 − x) ^n^ΔX(D)_A_ + x ^n^X(D)_B_ (1)

^n^ΔX(D)_eq_ = (δX_B_ − δX_A_) Δx (2)

^n^ΔX(D)_OBS_ = ^n^ΔX(D)_int_ + ^n^ΔX(D)_eq_ (3)

X could be ^13^C, ^1^H, ^15^N, ^19^F, etc. and x is the mole fraction of B. Δx is the change in the equilibrium upon deuteration.

**Figure 16:**
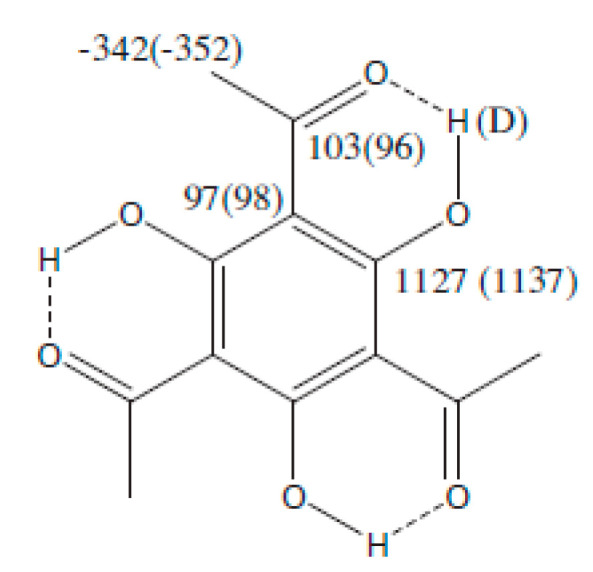
Deuterium isotope effects in CDCl_3_ + CD_3_OD and in brackets in CDCl_3_. The values are the sums of deuteration at all OH sites. Reprinted with permission from Ref. [44]. Copyright 2014 Elsevier.

This way, isotope effects on chemical shifts become a useful tool to establish whether or not tautomerism is present in cases in which this is not obvious. An example is 1,1′,1″-(2,4,6-trihydroxybenzene-1,3,5-triyl)triethanone (1,3,5-trihydroxy-2,4,6-triacetylbenzene). This was in CDCl_3_ shown not to be tautomeric based on deuterium isotope effects on ^13^C chemical shifts and DFT calculation of those [43]. This was further supported by low temperature studies showing that both the isotope effect and the OH chemical shifts were unchanged by lowering the temperature [44]. However, in ethanol this molecule was claimed to be tautomeric based on calculations [45]. This claim was investigated by the measurement of deuterium isotope effects on ^13^C chemical shifts in a mixture of CDCl_3_ and CH_3_OH and CD_3_OD, the latter in varying amounts. An extrapolation to 100% deuterium gives the isotope effects. A comparison of these with those in CDCl_3_ are shown in Figure 16. No real differences are found showing that no tautomerism takes place in methanol and by analogy not in ethanol.

**Figure 17:**
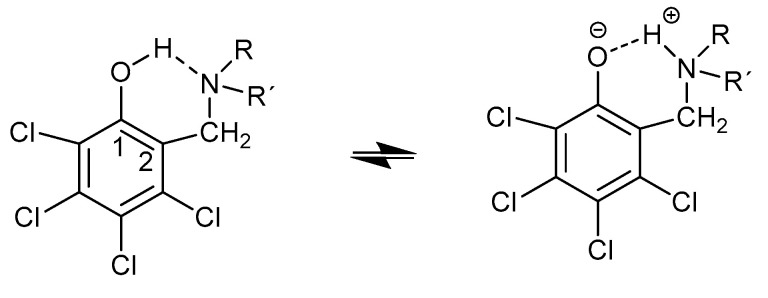
Tautomeric equilibrium of the Mannich base based on 2-hydroxy-3,4,5,6-tetrachlorobenzene.

Mannich bases are compounds that may or may not be tautomeric depending on the substituents at the aromatic ring. This is demonstrated in derivatives of 2-hydroxy-3,4,5,6-tetrachlorobenzene (Figure 17) as well as other derivatives. Temperature may also play a role [46,47].

**Figure 18:**
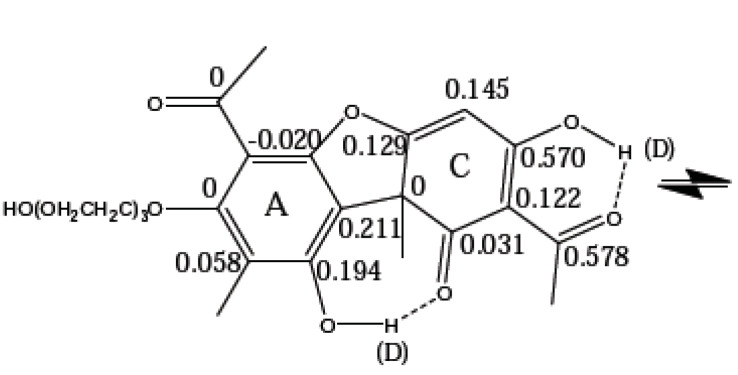
Deuterium isotope effects on ^13^C chemical shifts of a pegylated usnic acid. From Ref. [48].

Other tautomeric examples based on usnic acid are seen below. Usnic acid has important biological applications. However, it is rather insoluble. An attempt to make it more soluble is to add a pegylated side-chain as shown in Figure 18. Measurements of deuterium isotope effects on ^13^C chemical shifts showed that the equilibrium of the biologically important C ring is unperturbed [48].

**Figure 19:**
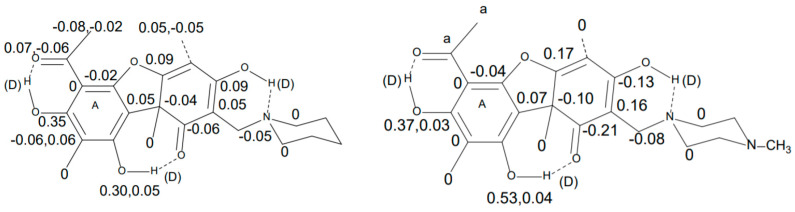
Mannich bases. Deuterium isotope effect on ^13^C chemical shifts. Top: In CDCl_3_. Similar effects are observed in a morpholine derivative. Bottom: In DMSO-d_6_. a. refers to the fact that the methyl group is partially deuterated so that the resonance is too complicated for analysis. The structure shown is only one of the tautomers. Reprinted with permission from Ref. [49]. Copyright 2018 Wiley.

Another derivative is the Mannich base derived from usnic acid as shown in Figure 19 [49].

**Figure 20:**
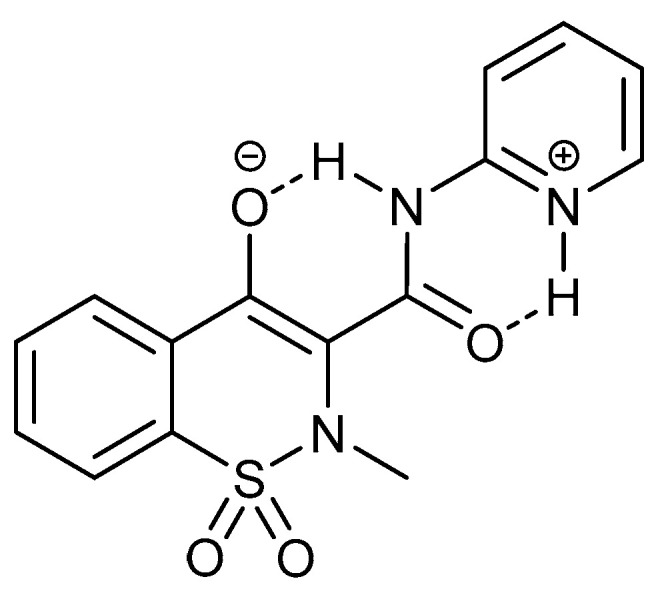
Zwitter ionic form of piroxicam.

Deuterium isotope effects on ^13^C chemical shifts in piroxicam showed that the addition of water shifted the equilibrium towards the zwitterionic form (Figure 20) [50].

**Figure 21:**
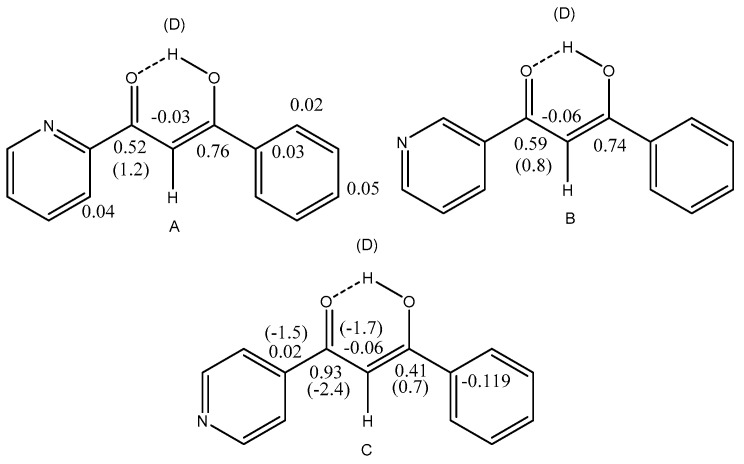
Deuterium isotope effects on ^13^C chemical shifts in the liquid and solid state. Only one tautomer is shown (see Figure 15). Numbers in brackets are from the solid state. Deuteration may also take place at C-2. Those isotope effects are small and do not tell us about the equilibrium and are left out for clarity. See Ref. [51].

Tautomerism may also occur in the solid state. An example is found in pyridoyl benzoyl β-diketones (Figure 21) [51]. In the liquid state, the two-bond deuterium isotope effects at C-1 and C-3 are slightly different reflecting that the equilibrium constant is slightly different from 1. In the solid state, the picture is very different (Figure 21). For **2** and **3** the effects are a clear sign of an equilibrium. However, for **1** a change in the crystal structure as a consequence of deuteration is suggested [51].

Changes in the crystal structure and the hydrogen bond structure is also discussed by Shi et al [52]. Ip et al. found in the triclinic phase a ^1^ΔN(D) of −2.7* ppm at 297 K. They concluded that deuteration of the XH proton of the complex between pentachlorophenol and 4-methylpyridine led a monoclinic structure with a weaker hydrogen bond than in the triclinic form [53].

**Figure 22:**
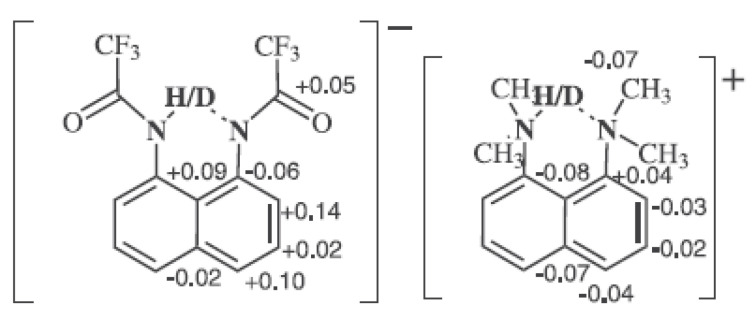
Deuterium isotope effects on ^13^C chemical shifts of a proton sponge. Reprinted with permission from Ref. [54]. Copyright 2013 Wiley.

Protonated proton sponges show both strong hydrogen bonds and tautomerism. The effect of the counter ion has been studied. In Figure 22, the counter ion is a proton-like sponge [54]. The effects in the proton sponge are similar to previous examples.

**Figure 23:**
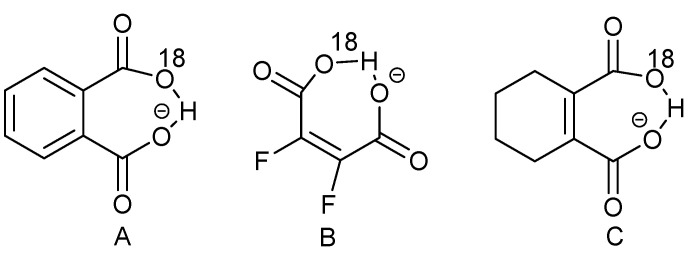
^18^O-labelled dicarboxylic acids. (**A**). phthalate monoanion; (**B**). difluoromaleimide; (**C**). cyclohexenedicarboxylic acid.

One of the questions in symmetric tautomeric systems with strong intramolecular hydrogen bonds is the position of the chelate proton. Two scenarios have been suggested: the chelate proton is jumping from one acceptor to the other or the proton is positioned at the center of the hydrogen bond. The difference being in a double-well potential or a single-well potential. For an early review see [55]. Based on calculations, Bogle and Singleton [56] proposed based that a coupling between a desymmetrizing mode and an anharmonic isotope-dependent mode could lead to isotope effects of the size found in, e.g., the phthalate monoanion (Figure 23A). In response to that, Perrin et al. studied ^18^O-labelled difluoromaleimide [57] (Figure 23B). ^19^F is a very chemical shift sensitive nucleus. It is thus very appropriate for the detection of small variations. The compound showed different chemical shifts for the two fluorines. This difference was related to a perturbation of the acidity of the carboxylic acid of the carboxylic acid due to the ^18^O substitution and not a simple isotope effect, as the dianion did not show chemical shift differences.

More recently, Perrin and Burke [58] found a temperature dependence of the C=O chemical shift of the ^18^O-labelled carboxylic acid of ^18^O-labelled cyclohexenedicarboxylic acid (Figure 23C). Based on this finding, they suggested an equilibrium.

In response to the paper by Bogle and Singleton, Perrin et al. [59] have studied ^18^O-labelled 1,2-cyclohexendicarboxylate monoanion (Figure 23C) as well as the difluoromaleate anion (Figure 23B). In both cases they found a larger isotope effect at a lower temperature, which is against a desymmetrization, as this should become smaller at a lower temperature.

**Figure 24:**
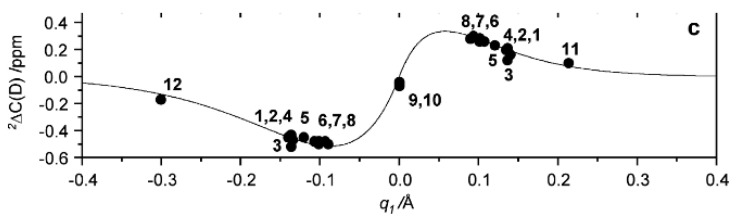
Plot of two-bond deuterium isotope effects on ^13^C chemical shifts of a series of primarily homoconjugated anions of carboxylic acids vs. q_1_. Data points for **11** and **12** are heteroconjugated dimers. q_1_ = 0.5(r_OH_ − r_HO_). Reprinted with permission from Ref. [60]. Copyright 2012 American Chemical Society.

Limbach et al. [60] studied deuterated maleate and phthalate anions (Figure 23A) as well as a series of homoconjugated anions of carboxylic acid (deuteration at the OH proton). The primary isotope effects are plotted vs. the two-bond deuterium isotope effects on ^13^C chemical shifts as seen in Figure 24. One can of course wonder why a distinction between a single-well and a double-well potential is so important, but this becomes clear when making plots such as the one in Figure 24. For maleate and phthalate, a single-well potential is assumed. Analysis of the data also allowed the construction of a rather complex correlation between ^2^ΔC(OD), q_1_ and three fitting parameters.

Succinic acid, *meso* and *rac*-succinic acid and methyl succinic acid with tetraalkylammonium ions as counter ions measured in CDF_3_/CDF_2_Cl at 300 to 120K showed double-well potential-based on isotope effects [61]. A plot of primary isotope effects vs. OH chemical shifts showed a large spread [62].

Carboxylic acid dimers have been studied trapped in a capsule. In case of partial deuteration, the encapsulation leads to slow exchange. The deuterium isotope effects on the OH chemical shift varies from 0.14 to 0.29* ppm for encapsulated acid vs. ~0.1* ppm for non-encapsulated acids. This can be related to the pressure of the encapsulation leading to a shorter O…O distance and a stronger hydrogen bond. Isotope effects correlate with OH chemical shifts [63].

**Figure 25:**
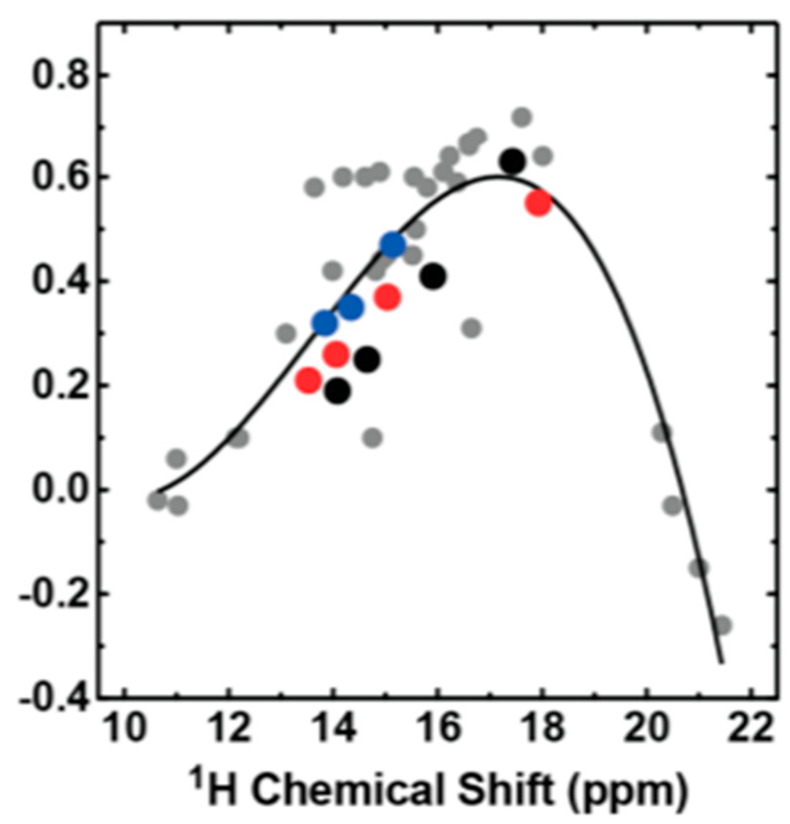
Plot primary deuterium isotope effects for a series of salicylates: 5-methylsalicylate, 4-methoxysalicylate, 5-formylsalicylate and 3,5-dinitrosalicylate. Chloroform (red), acetone (black) and water (blue) and for previously published O−H···O− hydrogen-bonded complexes in aprotic organic solvents (gray) [64] vs. OH chemical shifts. Reprinted with permission from Ref. [42]. Copyright 2015 American Chemical Society.

In Figure 25, a plot of primary deuterium isotope effects vs. OH chemical shifts for a series of primarily salicylates is shown [42]. This plot is very similar to plots in Refs. [3,62]. The change in the sign of the primary isotope effect was explained by Gunnarson et al. [40] finding a positive effect for weak double-well potentials and an increasing value as the anharmonicity increases. For those cases in which a single-well potential is the case, a negative value is found. As seen in Figure 25, the primary isotope effect for each compound is within 0.1 ppm in the different solvents showing that a solvent including water plays no special role for these hydrogen bonds.

## 5. Hydrogen Bond Energy

Hydrogen bond energies and the less well-defined hydrogen bond strength is clearly very important for molecular properties [65]. One way of getting a qualitative idea is the monitor isotope effects in solvents of different polarity. Sigala et al. [42] found no difference in going to water (see Section 4) and also for the pegylated usnic acid the isotope effects were similar in CDCl_3_ and in a mixture of water and DMSO [48].

**Figure 26:**
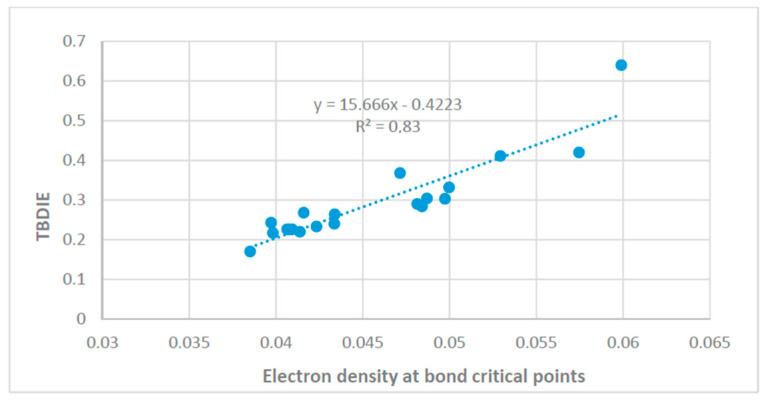
Two-bond deuterium isotope effects on ^13^C chemical shifts vs. electron density at bond critical points. Reprinted from Ref. [67].

Two-bond deuterium isotope effects on ^13^C chemical shifts may also be related to electron densities at bond critical points [66] (Figure 26). The latter are related to hydrogen bond energies.

**Figure 27:**
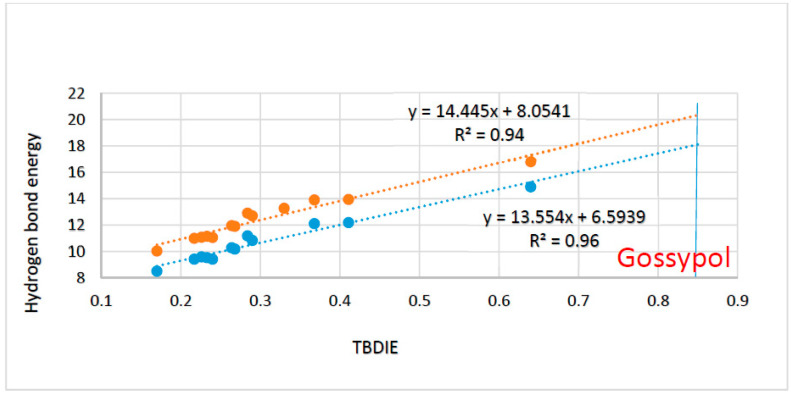
Plot of hydrogen bond energies vs. two-bond deuterium isotope effect on ^13^C chemical shifts of *o*-hydroxyacyl aromatics. Plot of ‘hb and out’ hydrogen bond energies in kcal/mol. calculated either with MP2/6-311++G(d,p) or with B3LYP/6-311++G(d,p) vs. observed two-bond deuterium isotope effect (TBDIE) on ^13^C chemical shifts in ppm. Top correlation line is B3LYP, bottom one is MP2. The observed TBDIE for gossypol is marked with the vertical blue line. Reprinted from Ref. [67].

A quantitative approach was proposed by Reuben [68], who correlated two-bond deuterium isotope effects with hydrogen bond energies of intramolecular hydrogen bonds. Recently, this was tested in *o*-hydroxybenzaldehydes (Figure 27) [67]. The hydrogen bond energies were calculated by the hb and our method. This method was originally formulated by Cuma, Scheiner and Kar [69]. Another example is found in 5-acyl rhodanines and thiorhodanines with bulky acyl groups [70] again with a very good correlation. Two-bond deuterium isotope effects are clearly a way of estimating hydrogen bond energies in cases in which the theoretical methods are less suited.

## 6. Correlations

Isotope effects can also be correlated to other parameters related to, e.g., hydrogen bond strength and hydrogen bond energy.

**Figure 28:**
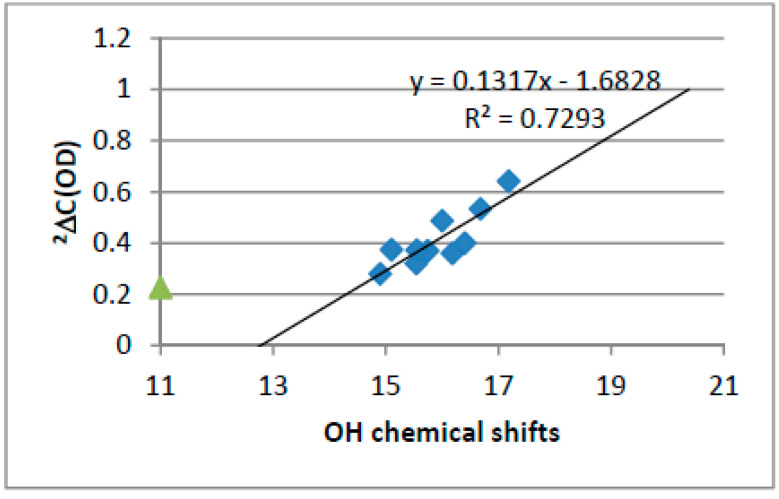
Plot of two-bond deuterium isotope effects vs. OH chemical shifts of 10-hydroybenzo[h]quinolones. Taken from Ref. [71].

Two-bond deuterium isotope effects on ^13^C chemical shifts are proportional to XH chemical shift. For an example see Figure 28. Another example is given in Ref. [71]. See also Figure 24 and Figure 25.

## 7. Theoretical Calculations

**Figure 29:**
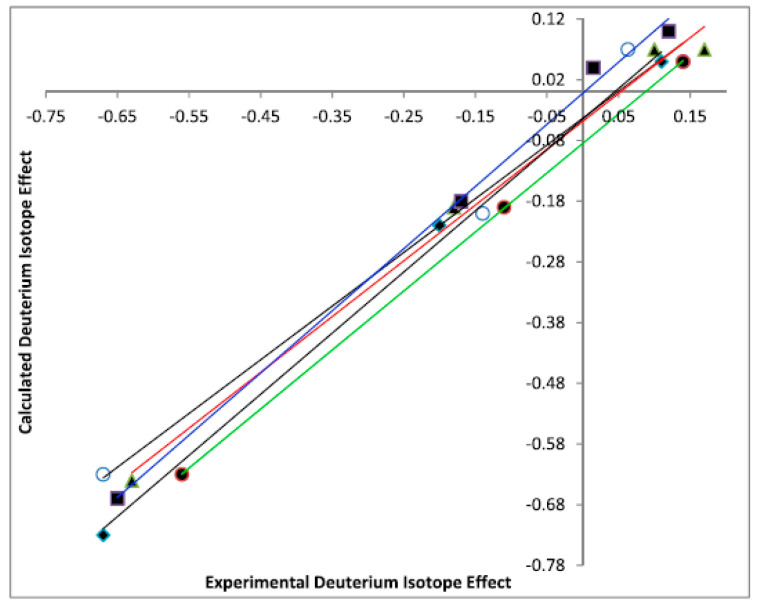
Plot of calculated vs. experimental deuterium isotope effects on ^13^C chemical shifts. The investigated compounds are of 
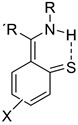
 type with R´ being methyl or phenyl and R being alkyl or aromatic. The calculations were of MP2/6-311+G(d2d,p) type. Reprinted with permission from Ref. [74]. Copyright 2018 Wiley.

A theory for the calculation of isotope effects on chemical shifts was presented by Jameson [72,73]. In a simplified way, deuterium isotope effects can be calculated by assuming that the XH bond is shortened upon deuteration. This approach has been described in a number of papers [62,71,72,74]. The shortening can be determined by the calculation of the hydrogen bond potential, but this is time consuming [43,75]. A simpler approach is to assume a reasonable value and as all the isotope effects of the molecule depend on the same shortening, a plot vs. experimental values will determine whether an intrinsic isotope effect is at hand. An example is shown in Figure 29.

An ab initio multi-component molecular orbital method on *o*-hydroxyacetophenones seems to overshoot the experimental values [76]. MC-MO calculations were also used for amino acid pairs [77]. A more recent example is a multicomponent hybrid density functional method combined with the polarizable continuum method [78]. This method was applied to picolinic N-oxide and led to decent predictions for the deuterium isotope effects on ^13^C chemical shifts, whereas the primary deuterium isotope effects were less well predicted. In the case of deuterium isotope effects, this was also the case for acetylacetone. Gräfenstein has developed a difference dedicated second-order vibrational perturbation theory to calculate isotope effects [79]. This was applied to a series of *o*-hydroxybenzaldehydes [80]. Ab initio path integral molecular dynamics (PIMD) calculations showed a barrier-less proton transfer and a C_2_V symmetry of the hydrogen bond. The calculated isotope effects were rather small [81]. An isocytosine dimer was studied at a low temperature. PIMD calculations were applied to the isocytosine base pair. For N3, the ^1^ΔN(D) = 0.88 ppm [82]. The PIMD calculations gave somewhat too large values, but of the right order.

## 8. Conclusions

Isotope effects on chemical shifts cover a very broad range of hydrogen bonds ranging from the very weak over dihydrogen bonds to the very strong hydrogen bonds found in, e.g., dicarboxylic acid anions. Isotope effects on chemical shifts in small molecules provide a basis and an understanding for use in larger, e.g., biological molecules. One-bond deuterium isotope effects on ^15^N chemical shifts and two-bond deuterium isotope effects on ^1^H chemical shifts can be used to monitor the distance to nearby hydrogen bond acceptors and/or charges.

Isotope effects on chemical shifts are a strong tool in the investigation of tautomeric systems and can lift degeneracies in symmetrical systems as seen in the monanions of dicarboxylic acids. ^18^O labelling of the latter is central in the discussion of single- vs. double-well potentials.

Two-bond deuterium isotope effects on ^13^C chemical shifts are related to hydrogen bond strength and to hydrogen bond energies.

## Data Availability

Not applicable.
